# Toward the future of OECD/ISO biodegradability testing-new approaches and developments

**DOI:** 10.1007/s00253-023-12406-6

**Published:** 2023-03-03

**Authors:** Uwe Strotmann, Gerald Thouand, Udo Pagga, Stefan Gartiser, Hermann J. Heipieper

**Affiliations:** 1 Department of Chemistry, Westfälische Hochschule, 45665 Recklinghausen, Germany; 2 Nantes Université, ONIRIS, CNRS, GEPEA, UMR 6144, 85000 La Roche sur Yon, France; 3 Rüdigerstr. 49, 67069 Ludwigshafen, Germany; 4 Hydrotox GmbH, Bötzinger Str. 29, 79111 Freiburg, Germany; 5 Department of Environmental Biotechnology, Helmholtz Centre for Environmental Research – UFZ, 04318 Leipzig, Germany

**Keywords:** Chemicals, Biodegradability, Standardized tests, OECD, ISO, Combined test systems, Biodegradation adaption potential (BAP), Inocula, Environmental biotechnology

## Abstract

**Abstract:**

In the past decades, industrial and scientific communities have developed a complex standardized system (e.g., OECD, ISO, CEN) to evaluate the biodegradability of chemical substances. This system includes for OECD three levels of testing (ready and inherent biodegradability tests, simulation tests). It was adopted by many countries and is completely integrated into European legislation (registration, evaluation, authorization, and restriction of chemicals, REACH). Nevertheless, the different tests have certain deficiencies, and the question arises of how accurately these tests display the situation in the real environment and how the results can be used for predictions. This review will focus on the technical advantages and weaknesses of current tests concerning the technical setup, the inoculum characterization, and its biodegradation potential as well as the use of adequate reference compounds. A special focus of the article will be on combined test systems offering enhanced possibilities to predict biodegradation. The properties of microbial inocula are critically discussed, and a new concept concerning the biodegradation adaptation potential (BAP) of inocula is proposed. Furthermore, a probability model and different in silico QSAR (quantitative structure-activity relationships) models to predict biodegradation from chemical structures are reviewed. Another focus lies on the biodegradation of difficult single compounds and mixtures of chemicals like UVCBs (unknown or variable composition, complex reaction products, or biological materials) which will be an important challenge for the forthcoming decades.

**Key points:**

• *There are many technical points to be improved in OECD/ISO biodegradation tests*

• *The proper characterization of inocula is a crucial point in biodegradation tests*

• *Combined biodegradation test systems offer extended possibilities for biodegradation tests*

**Supplementary Information:**

The online version contains supplementary material available at 10.1007/s00253-023-12406-6.

## Introduction

The estimation of the environmental behavior of chemical compounds has gained increased interest in the last decades. Many chemical products are marketed worldwide. Some basic data about the known chemicals are summarised in Table S1. From these data, it is obvious that a vast range of data is still missing. In addition, chemical pollution is regarded as a “planetary boundary,” and it may have an impact on other important global problems such as climate change and biosphere integrity (Rockström et al. 2009; Steffen et al. 2015; Wang et al. 2020, Diamond et al., 2015). Therefore, international directives and standards as well as national laws and norms have been issued to estimate the ecological behavior of chemicals in the environment.

Chemicals tested concerning their ecological behavior and especially their biodegradability are not only pure compounds, but also mixtures, some are known as UVCBs. They can be found in various forms ranging from daily products such as detergents, fragrances, and personal care ingredients to fuel and materials used in industry, e.g., for chemical reactions. UVCBs are produced worldwide and in very large amounts. Mentioned as an example are 1027 million metric tons of petroleum products, which were imported in 2018 into the European Union or manufactured in their member states. It is also estimated that about 20 to 40% of the chemicals registered in the USA and the European Union are UVCBs (Lai et al. 2022).

Another important group of products gaining increasing importance for their ecological behavior are synthetic polymers, which are usually very persistent in the environment. Prominent recent examples are micro-plastics in the aquatic environment, which may cause harmful effect on different organisms and even on whole ecosystems (Boots et al. 2019, Xu et al. 2020).

As a consequence of these facts, a profound knowledge of the biodegradability of chemicals is a crucial aspect to predict their environmental behavior. It seems logical that persistent chemicals may cause much more ecological problems than biodegradable ones. Since about 1960, standardization organizations and national states as well as industrial and scientific communities have developed and standardized many test methods and introduced complex test hierarchies (e.g., Organisation for Economic Co-operation and Development, OECD; International Organization for Standardization, ISO, Comité Européen de Normalisation, CEN) to evaluate the biodegradability of chemicals. Especially the OECD system is of great importance. It is based on three different levels and has been included in legislation by many European countries (registration, evaluation, authorization, and restriction of chemicals, REACH). Also outside of Europe, it is widely accepted. The importance of biodegradation tests is reflected in relevant publications and reviews (Painter 1995; Pagga 1997; Kaiser 1998; Vázques-Rodriguez et al. 2004; Kowalczyk et al. 2015; Gartiser et al. 2007, 2017, 2022 and 2023; Davenport et al. 2022). Although good test methods and test strategies are available, we must realize that they show technical deficiencies, and their boundary conditions are often arbitrarily fixed. Therefore, they have to be revised from time to time. Consequently, the question arises whether the current tests and test hierarchies represent the latest state of the art and how accurately they may reflect the behavior of chemicals in the real environment.

This review focuses on the advantages and weaknesses of the current tiered biodegradation test hierarchy and the included test methods concerning the technical setup, the characterization and adaptability of the microbial inoculum, and the use of adequate reference compounds. Several proposals on modern biodegradation tests including combined test systems and a discussion of biodegradation tests for UVCBs are presented. As an exemplary outlook of the importance of standardized tests in practical environmental biotechnological topics concerning remediation of soils and the degradation of plastics are also covered.

### Current biodegradation tests standardized by international and national organizations

Standardized biodegradation tests are usually used to predict the biodegradability of pure chemicals or single compounds in mixtures in defined environments, which are intended to simulate natural conditions to a certain degree. With an increasing level of simulation, the accuracy of the results obtained may increase, but the technical and analytical effort is also rising. In many cases, the use of simple test methods is sufficient to estimate, whether a compound will be biodegraded or not. Today, numerous test methods are available for this purpose. The choice of a certain test method usually depends on the intended purpose of the study and the physical-chemical properties of the chemicals. Especially the OECD and ISO have standardized a variety of biodegradation test, which are widely accepted in numerous countries. Particularly the OECD tests are of far-reaching importance as they often form the basis for regulatory measures. In this context, the OECD has created a three-tier system of biodegradability tests (ready biodegradability tests, inherent biodegradability tests, and simulation tests) which is widely accepted and of great importance.

### Ready biodegradability tests in the OECD system

#### Classical ready biodegradability tests

The first step in the tiered OECD system is the tests of “ready biodegradability” (RBT tests), which are summarized in Table 1. A substance is classified to be readily biodegradable, if it has reached a sufficient extent of biodegradation in one of the OECD 301 tests, the OECD 310 (2014) or the OECD 306 (1992) test (REACH guidance R.7b, ECHA (European Chemicals Agency) 2017b). The pass levels are 70% of DOC (dissolved organic carbon) removal compared to the start concentration, or 60% for BOD (biochemical oxygen demand) or CO_2_ production compared to a measured or calculated reference value, which has to be reached within a period of 10 days after the beginning of biodegradation, the so-called 10-day window. It has to be mentioned here that CO_2_ production and oxygen consumption (BOD) are very clear criteria for biodegradation, whereas DOC elimination only indicates the disappearance of carbon from the liquid phase. However, DOC measurements can also provide useful information on biodegradability if, for example, a clear lag phase is measured (the period from the start of the test to the onset of degradation), which can only be caused by biological processes. Physico-chemical processes such as hydrolysis may also potentially support it. In any doubtful cases, it is, however, helpful to use information from clear biological measurement parameters. Performing these tests it is not allowed to use an inoculum, which is pre-adapted to the test substance. Additionally, the ratio of biomass to carbon amount must be relatively low, and the maximum test duration is limited to 28 days. These test conditions are very stringent and are often criticized to be too strict and unfavorable if compared to natural conditions. Many chemicals that do not fulfill these criteria will nevertheless be fairly biodegraded in the environment (Pagga 1997, Painter 1995, Reuschenbach et al. 2003). Because of the stringent test conditions, consistent positive test results from valid tests generally supersede sporadic negative test results (see also REACH guidance R.7b, ECHA 2017a). When conflicting test results are reported, it is recommended to check the origin of the inoculum, as a possible adaptation of the inoculum might be the reason (OECD 2006). It should be known that the pass levels for complete biodegradation measured by BOD and CO_2_ production, in comparison to theoretical maximum values, are lower than those for DOC removal. The reason is that oxygen consumption and CO_2_ production start after the uptake of the carbon of the test substance into the bacterial cells and only that fraction can be measured, which is used for catabolic processes connected with energy production. The not detectable rest is assumed to be incorporated into new biomass. This can be expressed in terms of heterotrophic yield coefficients (Strotmann et al. 1999). The RBTs are often criticized for their artificial test conditions, but it is generally accepted that positive results are significant for the labels of “ready biodegradable” and “non-persistent.” If a negative test result is obtained, the criteria for ready biodegradability of a chemical are not met, and the substance is assumed to be potentially persistent. The persistence can, however, be confirmed or rejected within the test strategy by an improved test method (Gartiser et al. 2022 and 2023), for example, by the use of higher-tiered OECD simulation tests (OECD 307, 2002; OECD 308, 2002; OECD 309, 2004). These tests are conducted under more realistic environmental conditions, concerning, for example, the concentration of the test substance and the inoculum. In the course of these extended examinations, even the determination of degradation half-live times is possible, but this may require the use of radioactive (e.g., ^14^C) or stable isotope labeled (e.g., ^13^C, ^15^N) substances and the corresponding analytical equipment. Therefore, these tests are very cost-intensive and can usually only be performed if the industrial producers provide the test laboratories with labeled compounds.

**Table 1:** Established tests on ready biodegradability

Test	Method	Test principle	Remarks
DOC-die-away-test	OECD 301 A (1992), ISO 7827 (2010)	Static aerobic test system, measurement of DOC removal	Non-volatile water-soluble compounds
CO_2_ evolution test	OECD 301 B (1992), ISO 9439 (1999)	Static aerobic test system, measurement of CO_2_ production	Non-volatile water-soluble compounds
Continous CO_2_ evolution test	OECD 301 B (1992), ISO 9439 (1999)	Static aerobic test system, online measurement of CO_2_ production by conductivity measurement	Volatile/non-volatile water-soluble compounds, applied both as open and closed system (Strotmann et al. 2004)
Modified MITI (I) test	OECD 301 C (1992)	Static aerobic test, BOD determination, specific analysis possible	Non-volatile, water-soluble compounds;
Closed bottle test	OECD 301 D (1992), ISO 10707 (1994)	Static aerobic test, measurement of BOD	Volatile and toxic compounds at test concentrations of 2 mg L^-1^
Modified OECD screening test	OECD 301 E (1992), ISO 7827 (2010)	Static, aerobic test, measurement of DOC removal	Non-volatile water-soluble compounds at Low inoculum concentration
Manometric respirometry test	OECD 301 F (1992), ISO 9408 (1999)	Static, aerobic test, measurement of BOD, and comparison to COD and ThOD of the test substance	Poorly water-soluble, non-volatile, and volatile compounds
CO_2_ headspace test	OECD 310 (2014), ISO 14593 (1999)	Static aerobic test, measurement of CO_2_ evolution	Volatile compounds, comparable to the CO_2_ evolution test
Biodegradability in seawater	OECD 306 H (1992), ISO 16221 (2001﻿)	Static aerobic test system, measurement of DOC removal	Non-volatile water-soluble compounds,

#### Enhanced ready biodegradability tests

One of the declared aims of REACH is to accurately assess persistence in the environment and to reach this goal by certain enhancements to existing RBTs (ECHA, 2017a, b). Due to increasing criticism of the existing test methods in recent years, some enhanced RBTs (eRBTs) have been proposed (Gartiser et al. 2017; Gartiser et al. 2022, 2023). The aim is to close the technological gap between the classical tests on ready biodegradability and the much more complex simulation tests. The basic strategy for enhanced biodegradability testing is outlined in the ECHA guidances R.7b and R.11 (ECHA 2017a, b). At the moment, only two enhancements of the classical methods are allowed. These are a prolongation of the test duration up to 60 days which makes sense mainly for poorly water-soluble substances with low bioavailability and a slow but steady biodegradation behavior without reaching a plateau phase within the 28 days test duration, and additionally, the use of larger test vessels to achieve a higher probability that sufficient competent bacteria are present at the test start. A combination of these two modifications is still controversially discussed. The aim of these modifications is to provide more realistic test conditions and by doing this to obtain a more reliable prediction of biodegradability in the environment (ECHA 2017a, b).

#### Modified ready biodegradability tests

RBTs often show highly variable test results, and one striking disadvantage is false negative results, e.g., the test results are negative, but biodegradation in the environment will take place. This may have severe consequences for the labeling of chemicals when in the course of the evaluation the aim of ready biodegradability is not met. Therefore, certain modifications of the RBTs have been permitted, which concern issues such as bioavailability and microbial toxicity (OECD 301 annex II and III, Kowalczyk et al. 2015). For example, the use of bio-surfactants and humic acids is allowed to increase the solubility or miscibility in water of poorly soluble substances. ISO 10634 (2018) describes several methods for preparing poorly water-soluble substances for subsequent biodegradability testing. Moreover, lower test concentrations are allowed in case of an elevated toxicity under the test conditions. When all the other criteria of the RBTs are fulfilled, these modifications can be used for a better assessment of ready biodegradability.

#### Inherent biodegradability tests

The second step of the OECD test hierarchy are the so-called tests of inherent biodegradability (Table 2). The probability of a complete biodegradation in such an “inherent test” is much higher than in a “ready test” because these test systems are less stringent and have a higher degrading power. This is due to the higher and, therefore, more favorable ratio of test substance carbon to biomass. Chemicals that are biodegradable in an “inherent test” are regarded to be biodegraded under many natural and technical conditions (such as wastewater treatment plants, WWTPs) and are, therefore, classified to be non-persistent if some specific criteria are met (e.g., lag phase < 3 d, pass-level reached within 7 days (OECD 302 B, (1992) or 14 days (OECD 302 C, 1981). If, however, a chemical compound is not biodegraded in an “inherent test,” it is usually regarded to be persistent (Comber and Holt 2010). According to ECHA guidelines, a lack of biodegradation (< 20%) in an inherent test may be considered as evidence that the test substance is persistent without the need for further testing, while biodegradation above 20%, but below the pass level of 70% might be understood as evidence of primary biodegradability, suggesting that stable degradation products are likely to be formed (ECHA 2017a). The inherent tests are also often used to estimate the biodegradability of wastewater (ISO 9888, 1999) and even to determine biodegradation kinetics to predict their treatability in wastewater treatment plants. Experience shows that such test results are very credible. Therefore, these tests are not subject to the same criticism as RBTs. With respect to the compartment of wastewater treatment plants, they even show an increased practical implementation. The relevance of the test results may be improved by the use of higher inoculum concentrations than in RBTs. If the inoculum is directly withdrawn from the WWTP treating the industrial wastewater to be tested, instead of using the “weak” inoculum from a WWTP treating predominantly municipal wastewater, the results obtained are very realistic. It should be noted that currently no inherent test using the endpoint of CO_2_-evolution has been standardized, although this approach has been followed in several publications (Norr et al. 2001, Strotmann et al. 1995, Gartiser et al. 2017).

**Table 2:** Inherent biodegradability tests

Test	Method	Test principle	Remarks
Modified SCAS Test (Semi-continuous activated sludge)	OECD 302 A (1981), ISO 9887 (1992)	Semi-static, aerobic test system, fill- and draw method, measurement of DOC removal, test period up to 26 weeks	Non-volatile, water-soluble compounds, pre-adaptation and specific analysis to determine primary biodegradation possible
Zahn-Wellens/EMPA Test	OECD 302 B (1992), ISO 9888 (1999)	Static, aerobic test system, high test compound, and inoculum concentration, measurement of DOC removal	Non-volatile, water-soluble compounds
Modified MITI (II) Test	OECD 302 C (1981)	Static, aerobic test system, comparable to OECD 302 B (1992) but a specially prepared inoculum is required	Non-volatile, water-soluble compounds
Inherent biodegradability in soil	OECD 304 A (1981)	Static, aerobic test, addition of ^14^C labeled test compound, determination of ^14^CO_2_	Closed system; volatile/non-volatile and soluble/non-soluble compounds

#### Simulation tests

The third step in the OECD hierarchy are simulation tests which have the highest predictive power in the tiered system, because they are very close to environmental conditions (Table 3). They can simulate, for example, the biodegradation pattern in a biological WWTP rather well (OECD 303 A, 2001; OECD 314, 2008). Other simulation tests concern environmental compartments such as freshwater bodies of water and sediment (OECD 308, 2002; OECD 309, 2004) or marine surface water (see Table 3) and even anaerobic situations. Simulation tests are, however, often very costly and require a lot of analytical effort, time, and workforce. It is also difficult to automatize these tests. The major advantage lies in their high predictive value, because the test results are usually very close to the behavior of chemicals under real environmental conditions.

**Table 3:** Simulation tests

Test	Method	Test principle	Remarks
Aerobic sewage treatment A: activated sludge units B: biofilms	OECD 303 A (2001), OECD 303 B (2001)	Static, aerobic test system, measurement of DOC or COD decrease	Non-volatile, water-soluble, or dispersible compounds
Aerobic and anaerobic transformation in soil	OECD 307 (2002)	Static aerobic/anaerobic test, use of ^14^C labeled compounds, measurement of ^14^CO_2_ formation	Volatile water-soluble and poorly water-soluble compounds
Aerobic and anaerobic transformation in aquatic sediment systems	OECD 308 (2002)	Static aerobic/anaerobic test, use of labeled/unlabeled compounds, analysis of original compound, and transformation products	Non-volatile and slightly volatile compounds
Aerobic mineralisation in surface water	OECD 309 (2004)	Static/semi-continuous aerobic test system, use of labeled (^14^C)/unlabeled compounds, determination of primary/ultimate biodegradation	Non-volatile/slightly volatile compounds. water-soluble/poorly water-soluble compounds
Simulation tests to assess the biodegradability of chemicals discharged in waste water	OECD 314 (2008) A: Biodegradation in a sewer system OECD 314 (2008) B: Biodegradation in activated sludge test OECD 314 (2008) C: Biodegradation in anaerobic digester sludge test OECD 314 (2008) D: Biodegradation in treated effluent-surface water mixing zone test OECD 314 (2008) E: Biodegradation in untreated wastewater-surface water mixing zone test	Open/closed gas flow-through static systems, determination of primary/ultimate biodegradability, determination of transformation products, use of radiolabeled compounds recommended, but non labeled compounds permitted when an analytical procedure is given	All stages of wastewater treatment plant, volatile/non- volatile compounds, assessment of a mass balance

#### ISO standardized tests

Besides the test methods in the three-tiered OECD hierarchy, whereupon some of them have also been adopted by ISO or CEN, there also exist a number of other standardized biodegradation tests (Table 4). These have been standardized by ISO or CEN. Some of them have also been included in the REACH test method regulation (EU) No. 440/ 2008 or into OECD guidelines. It often happens that new test methods are developed in the ISO system and that they are later adopted by the OECD and implemented in REACH. This is the reason for the high conformity between ISO and OECD tests (see Tables 1, 2, 3, and 4).

**Table 4:** Other biodegradability tests and guidances

Test	Method	Test principle	Remarks
Anaerobic biodegradation test	OECD 311 (2006), ISO 11734 (1995)	Static, anaerobic test system, measurement of biogas production (CH_4_/CO_2_), test duration up to 60 days, inoculum: anaerobic sludge	Compounds in concentrations of 20 - 100 mg L^-1^ organic carbon
Aerobic composting test	ISO 14855-1 (2012) (general method), ISO 14855-2 (2018) (gravimetric method for the determination of CO_2_ evolution)	Static aerobic test system, use of an adsorbing material (Vermiculite) possible, measure-ment of CO_2_ production or oxygen depletion, extended test duration, higher test temperature	Solid polymeric compounds
Biodegradation of polymers in aquatic environment	ISO 14851 (2019) (oxygen depletion) ISO 14852 (2021) (CO_2_ evolution)	Static aerobic test system, measurement of CO_2_ production or oxygen depletion, medium with a higher buffer capacity, extended test duration	Miscible and water soluble polymeric compounds
Low concentration tests in water	ISO 14592 (2002)	Guideline to perform biodegradation tests at very low concentrations	
Guidance for poorly water-soluble compounds	ISO 10634 (2018)	Guideline to perform biodegradation tests with poorly water-soluble compounds	
Guidance for selection of biodegradation tests	ISO 15462 (2006﻿)	Tests in the aquatic environment	

#### Anaerobic biodegradation tests in ISO and OECD tests

In contrast to aerobic biodegradation processes, the anaerobic breakdown of organic compounds occurs under oxygen-free or at least under reducing conditions. The process itself is rather complex and can be divided into four subsequent stages (Gottschalk 1986), but for testing purpose only the end products methane and carbon dioxide are relevant. The anaerobic biodegradability test (ISO 11734, 1995;  OECD 311, 2006) models the “ultimate” biodegradation in digesters of municipal WWTPs. This test method is useful for compounds, which are not degradable under aerobic conditions but adsorb onto activated sludge flocs, which are finally digested in an anaerobic reactor.

The gaseous end products are measured via the increase of pressure in the headspace of the test vessels. They contain an appropriate inoculum from an anaerobic digester, a mineral salt solution, the test compound, and a reference compound (sodium benzoate, phenol, or polyethylene 400 are recommended). The test duration is up to 60 days and the test temperature is at 35 °C (ISO 11734, 1995; Pagga and Beimborn 1993). A comprehensive survey of the anaerobic biodegradability of different classes of organic compounds has been published by Battersby and Wilson (1989).

Furthermore, the method can also be used for biological waste, which is treated in anaerobic treatment plants. Also, highly contaminated wastewaters with a high load of organic substances, measured as total organic carbon (TOC) or chemical oxygen demand (COD), are often treated in high-load one- or two-stage anaerobic reactors. Strategies with a first step based on the ISO 11734 (1995) method and a subsequent laboratory-scale fixed bed reactor test to evaluate the anaerobic biological treatment of wastewaters for anaerobic degradation have already been developed and are successfully used in practice (Strotmann et al. 1993b). Moreover, these test results are very valuable for the basic layout of industrial anaerobic wastewater treatment plants (Abwassertechnische Vereinigung ATV-FA 7.5 1994, 2004).

#### Biodegradation of polymers in ISO tests

Concerning the biodegradation of polymers, there exist two principles of standardized test methods, which are based on the depletion of oxygen (BOD measurement) and the evolution of carbon dioxide. One test target is focused on polymeric compounds in aquatic environments ISO 14851 (2019) based on OECD 301 F (1992) and ISO 14852 (2021) based on OECD 301 B (1992) and the other in a controlled composting test system (ISO 14855 part 1 (2012) and ISO 14855 part 2 (2019)). When regarding the evolution of carbon dioxide, it is emphasized that the buffer capacity in an aquatic test system has to be high enough, because high amounts of carbon dioxide can be evolved during the prolonged test period. Other critical items are the nutrient supply and the use of an appropriate inoculum (Pagga et al., 2001; Pagga et al., 1995; ISO 14851 (2019) and ISO 14852 (2021)).

Possible reference compounds in the aquatic system are aniline, microcrystalline cellulose, or ß-hydroxybutyrate. As an inoculum, activated sludge containing 10^3^ to 10^6^ CFU/mL (colony-forming units) derived from a municipal WWTP is recommended. If possible, also the determination of a flow of carbon of the test compound (carbon balance) should be performed to gain more information on biodegradation.

When using a composting test system vermiculite may be added as an adsorbing substrate. The inoculation occurs through an aqueous compost extract. In order to ensure sufficient nutrients in the system, a mineral salt solution and a mixture of trace elements are added. The test duration is up to 6 months, and the incubation temperature is 58 °C. As a reference material, TLC (thin layer chromatography cellulose) is recommended (ISO 14855 part 1 (2012) and ISO 14855 part 2 (2018)).

### Critical items and new approaches for biodegradation tests

#### General critical points

Biodegradation tests in connection with chemical testing have been criticized since they have been published. However, it has to be kept in mind that biodegradation is only part of the information needed to predict the environmental fate of chemicals. Other important aspects are the physico-chemical features such as log P_ow_, solubility in water, adsorption to different kinds of materials, volatility, and the ecotoxicological effects on various test organisms of different trophic levels. However, biodegradation is a fundamental property of a chemical, because a substance that is degradable will usually cause fewer ecological problems than a persistent one. A crucial factor is the composition of the used microbial inoculum, which may underlie great fluctuations. Therefore, this parameter is difficult to be perfectly simulated in biodegradation tests. Furthermore, most RBT are performed under unrealistic environmental conditions with relatively high concentrations of the test chemicals compared to a relatively low inoculum concentration. Thus, simple standardized tests can never represent various real conditions with their much higher biodegradability potential. Other items are the test conditions which have been standardized and which do not represent real conditions and which may vary considerably (Lapertot and Pulgarin 2006). Other limitations normalized by both OECD and ISO and regulated by ECHA concern the pass levels and the test duration, as well as the exclusion of pre-adaptation (pre-exposure) of inoculum to the test chemical. RBTs are generally criticized to produce false negative results due to their unrealistic high level of stringency. Some of these concerns can be refuted by choosing an appropriate test method and optimizing the relevant parameters within the allowed frame. However, the authors see the urgent need to modify and improve the OECD test guidelines 301 and 302 under consideration of the experiences obtained since the last revision in 1992.

#### Sources of inocula and their characterization

Regarding biodegradation processes, microorganisms and especially bacteria in the used inocula play the most important role. For this reason, the source of inoculum is a very important factor. Inocula can be taken from activated sludge, sewage effluent, soils, or surface waters. Details are described in the test methods. Due to their different origin, the cell densities can vary in a wide range reaching from, e.g., 10^4^ cells L^−1^ in the closed bottle test (OECD 301 D, 1992) to 10^8^ cells L^−1^ in the OECD 301 A/B/C/D/E/F (1992) tests. In inherent biodegradability tests such as the Zahn-Wellens test (OECD 302 B, 1992), the cell concentration is much higher and can be about 3*10^9^ cells L^−1^ (at a suspended solids (SS) conc. of 1g L^−1^). Another important factor is that the concentration of bacteria which have specific degradation properties (competent cells) is much lower than the total concentration of bacteria. The concentration of competent cells is estimated to be only in the range of 2.5 * 10^4^ cells L^−1^ to 3.7 * 10^6^ cells L^−1^ (Kowalczyk et al., 2015, Thouand et al., 1995, 2011). Investigations have shown that about 10^5^ competent cells L^−1^ are needed to degrade a reference compound such as 4-nitrophenol (Thouand et al., 1996). In addition, the adaptation of inoculum is a crucial factor because a microbial community may fluctuate concerning its composition or biochemical activity under the selective pressure of a test compound. The reason is that bacteria with the potential to degrade a specific compound grow better and faster and enable effective biodegradation of this compound. The period up to the visible onset of biodegradation, usually defined as the level of 10% degradation, is designated as lag-phase or lag-period based on the usual biotechnological terms of bacterial growth. The lag-phase in biodegradation tests can vary from less than 1 to about 20 days or more depending on the compound tested and is often very characteristic and reproducible. Strotmann et al. (1993a) showed that for morpholine the lag period in ready tests, inherent tests, and even in laboratory wastewater treatment plants usually is at about 14 days, but it can be shortened to only 1 to 2 days when a pre-adapted inoculum is used. However, according to the OECD test guidelines pre-adaption of the inoculum is not allowed, because the primary aim is to make general predictions and not to model specific situations. The clear positive effect of pre-adaptation is described in several publications (Spain and van Veld, 1983; Thouand et al., 1996; Strotmann et al. 1993a).

In contrast to pre-adaptation, acclimation processes of the inoculum to the test conditions may include pre-conditioning (aeration for 5 to 7 days, OECD 301) or inoculum preparation procedures (filtration, centrifugation, settling and decantation, aeration and incubation of the inoculum with glucose and peptone in the modified MITI (I) test, (OECD 301 C, 1992c)). The main purpose of these procedures is to lower blank values including a better signal to noise ration and also a homogenization of the inoculum. These different processes aim at achieving a better measurement of biodegradation. It has to be stressed that in contrast to pre-adaptation, during acclimation processes the inoculum does not have any contact to the test substance. However, it was also found that during acclimation, the inoculum tends to lose some biodegradation potential. After such an acclimation, some inocula can even no more degrade widely used reference substances such as aniline that is classified to be readily biodegradable. Therefore, the benefit of these procedures in biodegradation tests is controversially discussed (Vázquez-Rodriguez et al. 2007; Goodhead 2014).

The effect of a pre-treatment of the inoculum is for example obvious when the so-called modified MITI (I) test (OECD 301 C, 1992) is compared with the original version of the Japanese MITI biodegradability test. For the latter, an inoculum is used which is pre-cultured with synthetic sewage. The biodegradation potential of this inoculum is much lower than that of the modified MITI (I) test (OECD 301 C, 1992), where activated sludge from normal municipal WWTPs is permitted (Kayashima et al. 2014, Nabeoka et al. 2016).

In order to use an active inoculum, the intrinsic metabolic potential of the microorganisms and, therefore, also the general biodegradation potential can be estimated by various methods, for example, by measuring respiration and dehydrogenase activity as well as the profile of hydrolytic enzymes and the concentration of total, active and cultivable cells (Vázquez-Rodriguez et al. 2003, 2011). These parameters may provide insight into the intrinsic metabolic activities of the inoculum, but they are laborious and it is difficult to transfer data from non-adapted to pre-adapted inocula.

Another possibility to characterize various inocula could be a categorization into different classes due to their ability to degrade model compounds after a certain adaptation period (lag phase). This lag phase may indicate the biodegradation adaptation potential (BAP) of an inoculum fairly well. The reference substances that are used in every test need to have certain properties to deliver useful information. They should not be biodegraded too fast but need to show a clear level of biodegradation within the test duration. Class I compounds (low BAP) have a lag period in the range of 0 to 2 days, class II compounds (medium BAP) have lag periods of > 2 to 5 days, and class III compounds (high BAP) need more than 5 days until biodegradation starts. A compilation of such data can be found in Table 5. In this way, an intrinsic biodegradation capability of inoculum may be evaluated. It could be claimed that an inoculum should have at least a BAP of class II to be used in a biodegradability test. However, this concept should be further evaluated in ring tests before it can be introduced into standards.

**Table 5:** Possible compounds for assessing the “Biodegradation adaptation potential” (BAP) of an inoculum based on different lag periods before the onset of biodegradation. Class 1: low BAP , lag period: 0 to 2 days; class 2: medium BAP, lag period: > 2 to 5 days; class 3:  high BAP, lag period: > 5 days

Compound	Test system	Extent of degradation (%)	lag period (d)	Reference
**Class 1**				
Benzoate	OECD 301 F (1992)	91–93	0	Reuschenbach et al. 2003; Gu et al. 2021
Acetate	OECD 301 F (1992)	< 95	0	Gu et al. 2021
Glycerole	OECD 301 F (1992)	68–78	1	Reuschenbach et al. 2003
Cyclohexanone	OECD 301 F (1992)	83–96	0.5	Reuschenbach et al. 2003
2-Ethylhexylacrylate	OECD 301 F (1992)	58–73	1	Reuschenbach et al. 2003
**Class 2**				
Aniline	OECD 301 F (1992)	75–86	3.4–3.8	Strotmann et al. 2004; Strotmann et al. 1995
Acrylic acid	OECD 301 F (1992)	91–94	2.5–3	Reuschenbach et al. 2003
Phenol	OECD 301 F (1992)	76–81	3	Reuschenbach et al. 2003
1.5-Pentanediol	OECD 301 F (1992)	83–89	3	Reuschenbach et al. 2003
4-Isopropylphenol	OECD 301 F (1992)	70–80	2.3–5	Comber and Holt 2010; Gu et al. 2021
**Class 3**				
NTA	OECD 301 F (1992)	70–92	9–16	Reuschenbach et al. 2003; Strotmann et al. 1995
Morpholine	OECD 301 F (1992)	87–89	16	Reuschenbach et al. 2003
2- Chloroethanol	OECD 301 F (1992)	93	5–6	Strotmann et al. 1995
Diethylene glycole	OECD 301 F (1992)	62–90	5–8	Strotmann et al. 1995; Gartiser et al. 2022
Ibuprofen	OECD 301 A/B (combination test)	98*	12	Gartiser et al. 2022
4- Fluorophenol	OECD 301 A/B (combination test)	73*	9	Gartiser et al. 2022

*Based on CO_2_ production

Investigations have shown that different activated sludges collected worldwide differ very much in their activity and microbial diversity (Forney et al. 2001, Prosser et al. 2007). But there are also studies that show that independent from the inocula used, the microorganism community structure in aerobic sludge systems operated under the same conditions are similar (Muñoz-Palazon et al. 2018). Anyway, the origin of the sludges (municipal or industrial WWTP) has a great influence on the test results. It is obvious that an inoculum with a high bacterial diversity and, therefore, a higher biodegradation potential for many chemicals will have a much higher chance to contain competent species, which can degrade a specific test substance than an inoculum with a lower diversity can do. In addition, the amount of inoculum added or the volume of the test batches plays a decisive role (Kowalczyk et al., 2015; Painter, 1995). The great importance of the concentration of the inoculum was investigated and described already in the early phase of the development of the existing test methods (Haltrich et al. 1980)

#### The concentration of test chemical and sludge loading in the test systems

Test chemicals can have a stimulating, but also an inhibitory effect on biodegradation as a review by Painter (1995) with a compilation of detailed examples has shown. In this study, data from several OECD tests and field studies are listed indicating that the concentration of a chemical plays a crucial role concerning the lag period and the extent of biodegradation. Compounds investigated have been 4-nitrophenol, several surfactants, phthalates, chlorinated compounds, phenol, benzoates, benzylamine, and the herbicide 2.4-dichlorophenoxyaceticacid (2, 4-D). As a conclusion, it is recommended that the concentration of the test chemical should generally be chosen as low as possible. It is also recommended that, in the case of inhibiting chemicals, the test concentration should be at about 10% of the EC_50_ value as measured in a standardized microbial toxicity test. Such tests are discussed by Strotmann et al. (1992, 1993, 2020). A compilation of relevant OECD and ISO guidelines for bacterial toxicity is given in Table S2 and the annex II of OECD 301. Examples for the biodegradation of toxic compounds are given by Nabeoka et al. (2016), who lowered the concentration of several test compounds in OECD 301 C (1992) tests from 100 mg/L to 30 mg/L and could increase the biodegradation of sodium dimethyldithiocarbamate, 4-chloro-3-cresol, thymol and p-tert-butyl-α-methylbenzenepropionaldehyde in this way. A similar problem may arise when biocides are tested which exhibit an intended toxicity.

Furthermore, O’Malley (2006) conducted a study, where he tested the lowest possible test concentration of the model substance sodium acetate in the OECD 301 F (1992) test (respirometric test). He reported certain problems such as biphasic growth curves when using activated sludge as an inoculum. The problem could be solved when using an alternative inoculum that was composed of a consortium of bacteria isolated from activated sewage. Here, he could lower the dosage concentration from 100 to 2.5 mg/L, which lies in the same range as in the “Closed Bottle test” (OECD 301 D, 1992). On the other hand, the standardization of inoculum is criticized by Forney et al. (2001) and Pagga (1997) who argue that standardized inocula will lead to a decrease in microbial diversity and a loss of biodegradation potential. On the contrary, some authors even favor the usage of commercial mixtures of bacteria (Paixão et al. 2000, 2003; Tabka et al. 1993), arguing that the quality of natural inocula will vary from source to source whereas a defined mixture always has the same composition. However, the result may be that these inocula invariably have the same artificial biodegradation potential.

Low concentrations of test compounds can cause a longer adaptation period in comparison to higher concentrations (Berg and Nyholm 1996). It was also found that certain threshold concentrations are necessary to start biodegradation because no adaptation occurred at concentrations below these threshold values (Efroysmson and Alexander 1995). The reason for this phenomenon may be that microorganisms need sufficient time and minimum concentrations of the substrate to induce genes and synthesize enzymes necessary for degradation and growth (Grady 1984).

Predicting biodegradation under realistic environmental conditions, especially at very low concentrations, can require simulation tests, which are very laborious and expensive. Koziollek et al. (1996) described a dynamic river model and tested its usefulness with aromatic compounds. This work finally led to ISO 14592 (2002) describing biodegradation at low concentrations in water. Pagga 1987 had previously discussed the problem of unrealistic high concentrations of test substances in biodegradation tests compared to those occurring in the natural environment.

The sludge loading is closely connected to the test chemical concentration. This term originates from wastewater technology, and it describes the ratio of wastewater (e.g., measured as dissolved (DOC) or total (TOC) organic carbon), to the amount of activated sludge in a treatment plant, measured as suspended solids (SS). This term is equivalent to the food-to-biomass (F/M) ratio. In the OECD 301 A (1992) and OECD 301 F (1992) tests, the initial sludge loading ranges from 0.33 to 1.33 g DOC g^−1^ SS. The initial sludge loading in the OECD 301 E (1992) test is higher ranging from 3.3 to 13.3 g DOC g^−1^ SS due to a very low inoculum concentration. As a comparison, in an inherent biodegradability test such as OECD 302 B (1992) test the initial sludge loading is in the range of 0.2 to 0.4 g DOC g^−1^ SS.

As shown for the reference compound diethylene glycol in a combined CO_2_/DOC test system (Strotmann et al. 1995) with a fixed DOC concentration of 40 mg L^−1^ and a variable inoculum concentration (range: 5 to 500 mg L^−1^), the shortest lag periods and the highest biodegradation rates were observed at the highest inoculum concentrations which correspond to the lowest sludge loading. Furthermore, it could be shown that a minimum inoculum concentration of 100 to 200 mg SS L^−1^ was necessary to enable the degradation of diethylene glycol within the normal test period of 28 days. This finding could also be confirmed in an OECD 302 B (1992) test. The reason for this observation probably lies in the absolute mass of the inoculum added. The higher the absolute mass of inoculum is at a given DOC concentration, the smaller is the initial sludge loading. This results in a better adaptation and a shorter lag period. Consequently, it is recommended to improve the reliability of RBTs by an increase of the mass of inoculum, which has to be explicitly mentioned when performing standardized tests.

#### Test volume

The positive effect of higher initial inoculum biomass is also the reason to increase the test volume of a test system to enhance adaptation processes (Gartiser et al. 2022). Because in RBTs low-density inocula are used, the chance to have sufficient “competent degraders” in the test is diminished (Thouand et al. 1995, Ingerslev and Nyholm 2000; Ingerslev et al. 2000). Low test volumes in RBTs may decrease the likelihood of variations in microbial diversity, and as a consequence, there is a high probability to get large fluctuations in test results (Nyholm et al. 1992). An increase in the test volume may enhance the diversity of the microbial population/biocenosis and may allow better adaptation processes and, therefore, an improved reproducibility of test results.

#### pH

The media used in ISO and OECD tests have on average a phosphate concentration of only 3.7 mM, resulting in rather poor buffering properties. In a combined test system (Strotmann et al. 1995), the phosphate content was raised up to 25.1 mM to enable stable buffering properties in the range of pH 7.0, which is needed for biodegradation processes. Such high phosphate concentrations and buffering capabilities are unlikely to take place in waters under natural conditions, but they are necessary to enable a stable CO_2_ release in test systems. A pH shift may for example occur when organic acids or their salts are biodegraded. This may also occur during the biodegradation of certain polymeric substances and the hydrolytic dehalogenation of halo-organic compounds (Janssen et al. 1985; Strotmann et al. 1990). Many authors showed that the release of CO_2_ strictly depends on the pH of the medium (Struijs and Stoltenkamp 1990; Goss et al. 2020; Larson and Games 1981; Larson and Payne 1981; Painter 1995). Therefore, a stabilization of the pH by adequate buffering is necessary to obtain reliable test results. This should be considered when standardized tests are re-evaluated and modified.

#### Compounds poorly soluble in water

Compounds that are poorly soluble in water may exhibit certain difficulties when used in DOC-based OECD screening tests, which require a DOC concentration in the test vessel of at least 10 mg L^−1^. The only exception is the closed bottle test (OECD 301 D, 1992) with a test concentration in the range of 2–10 mg L^−1^ which is equivalent to a DOC concentration of about 0.8 to 4 mg L^−1^. According to the general introduction of the OECD 301 guideline, test methods based on the endpoints CO_2 _evolution or oxygen depletion such as the OECD 301 B (1992) or OECD 301 F (1992) tests may also be applied for poorly soluble substances. One possibility to overcome these difficulties would be the performance of cost-intensive simulation tests using ^14^C labeled compounds. Other possibilities are the use of dispersion techniques, like direct addition of the test compound, the application of an inert absorbent, ultrasonic treatment, or dispersion via emulsifiers (Nyholm 1990). In this study, different application methods were tested in a respirometric system, and it was found that no dispersion technique was universally the best for the test compounds anthraquinone and di-isooctylphthalate. For liquid compounds, an application via a volatile organic solvent (dichloromethane) or a silica gel was possible, whereas the direct application and the use of emulsifiers led to difficulties. On the other hand, Richterich et al. (1998) developed a two-phase-closed bottle test, which is based on the classical closed-bottle test OECD 301 D (1992) with an increased headspace and, therefore, a bigger reservoir for oxygen. In this system, an increased inoculum concentration of 30 mg L^−1^ activated sludge may be used, instead of the much lower inoculum concentration of the original close-bottle test (5 mL L^−1^ effluent of a WWTP, which is equivalent to about 1.5 mg SS L^−1^). The need for testing poorly soluble, hydrophobic compounds has led to the proposal of a further “inherent” biodegradability test (Battersby et al. 1999). The present method (ISO 14593, 1999) is based on a CO_2_ headspace test (Strujs and Stoltenkamp 1990, Battersby 1997), where a pre-exposed inoculum is used and the test duration is extended up to 56 days. In a ring test, several hydrophobic oil products have been tested, and the results showed that it is a practical method which should be considered as well by the OECD to be integrated into the test guidelines (Battersby et al. 1999). Another possibility would be to use combined test systems or a multicomponent test, which would offer more analytical possibilities, for example, by using specific analytical techniques or the analysis of the total carbon flow. The problems that arise during testing the biodegradability of poorly water-soluble test compounds and suggestions for their treatment are summarized in ISO 10634 (2018)—guidance for poorly water-soluble test compounds.

Synthetic polymers represent a special group of substances, which are not or only poorly dissolved in water. Many of them are not biodegradable and, therefore, very stable after use when they are released into the environment. This leads to immense problems in the environment and was one of the reasons to develop biodegradable or at least compostable polymers (Pagga et al. 1996). The European packaging material regulation also had a major influence on the development of biodegradability test methods for polymeric materials. Details of the method development were described by Pagga (1998, 1999) and have meanwhile been implemented into numerous ISO standards specifically adopted for testing polymers (see Table 4).

#### Reference compounds

In the OECD tests and ISO standards, a number of reference compounds are used to determine the quality of the inoculum and to ensure that tests have been correctly performed. Different reference compounds have also been critically discussed in the literature (Gu et al. 2021; Kowalczyk et al. 2015; Painter, 1995; Kaiser, 1998). Comber and Holt (2020) published a detailed list of reference chemicals and proposed a so-called bin-system to classify them according to their behavior in RBTs (Table S3). The focus was mainly put on the extent of biodegradation, but also the kinetics of the degradation processes is considered. In Table S4, detailed biodegradation data of the most important reference compounds are given. Some reference compounds such as benzoate and acetate are, however, not suitable, as they degrade too fast. They are even said to be degraded without any inoculation (Gu et al. 2021) and do, therefore, not deliver significant degradation curves.

A new application of the use of reference compounds could be the estimation of the biodegradation adaptation potential (BAP) of an inoculum. In this case, only class II or class III compounds (see Table 5) should be chosen. An important prerequisite is the reliable assessment of biodegradation kinetics to determine the lag period. For this purpose, suitable reference compounds are aniline (class II), 2-chloroethanol, and diethylene glycol (class III). 2-chloroethanol offers the advantage that biodegradation kinetic may be followed by the production of carbon dioxide, the consumption of oxygen, the removal of DOC, and even the liberation of chloride anions (Janssen et al. 1985; Strotmann et al. 1990).

### Measurement of biodegradation: the quest of the Grail

#### Current technical possibilities

Evaluating the biodegradation of a single compound or a complex mixture of organic molecules is a process intimately linked to the ability to follow at least one parameter of the biodegradation equations below 
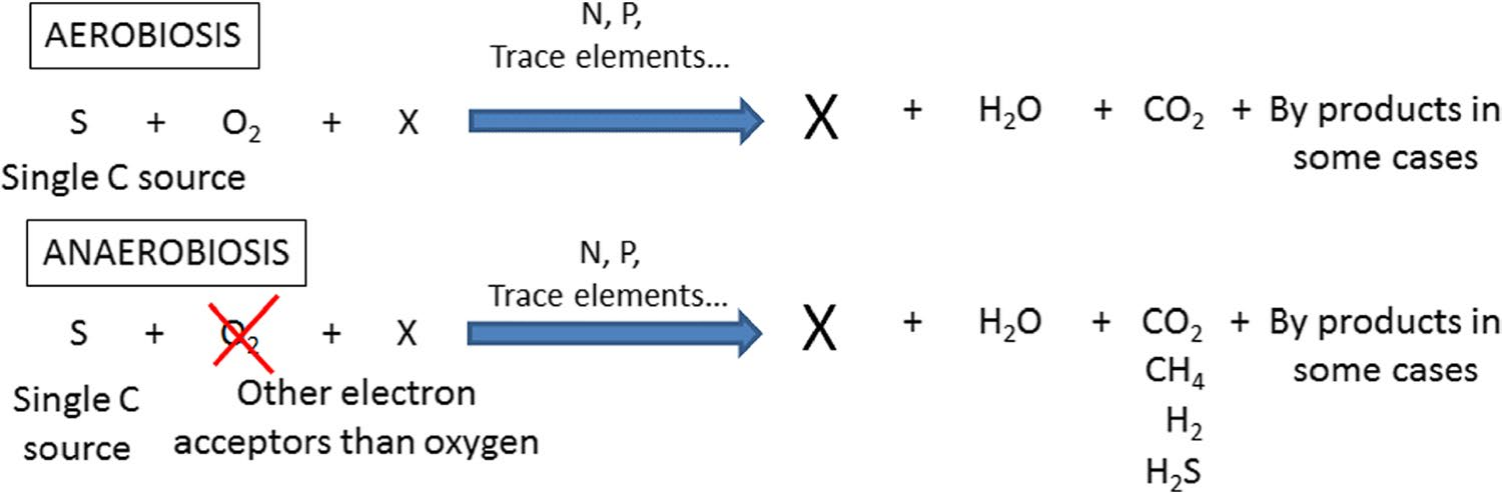


Basically, the usual methods for the assessment of biodegradability can be divided into two groups. The methods of the first group make use of the direct measurement of the disappearance of the organic parent compound by specific analysis to gain information about the so-called primary biodegradation. The methods of the second group use indirect measurements of the bioconversion of the parent compound, such as carbon dioxide production, a decrease of dissolved organic carbon (DOC), and oxygen consumption (BOD). The measurement of these indirect parameters is used to determine the so-called ultimate biodegradation. They are easier to perform than specific analysis and can be automated more easily. The measurement of dissolved organic carbon (DOC) remains subject to sampling and measurement by a carbon analyzer, as recommended by OECD 301 A (1992). At present, there are no automated systems that can solve the difficulties caused by the filtration of the sample to estimate the dissolved part of the carbon. Other parameters such as the oxidation of the test chemical and, moreover, for UVCBs chemical mixtures compromise the design of an automated instrument (Chow et al. 2022).

**Fig. 1:**
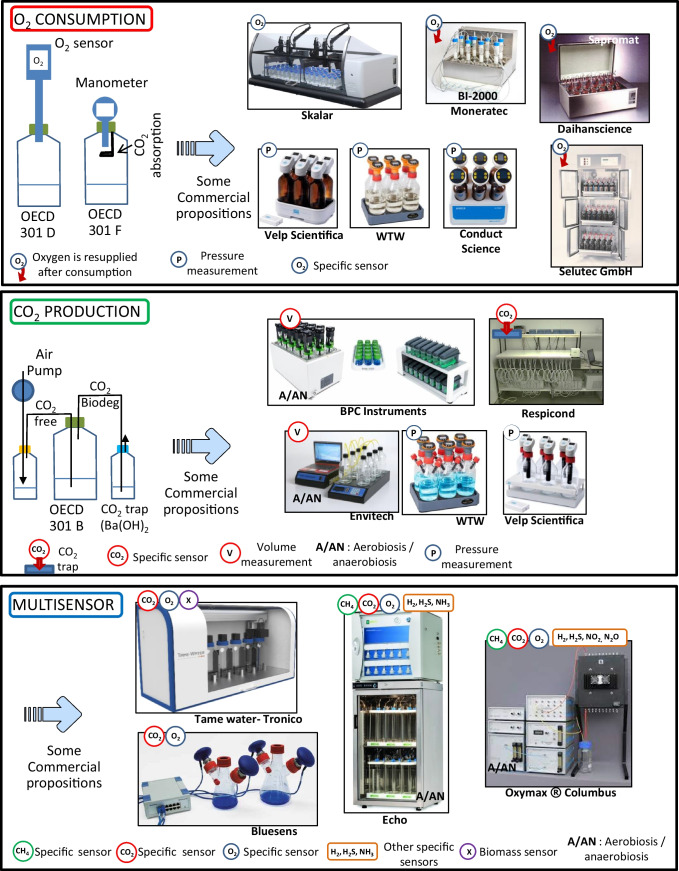
Principle of the OECD 301 D, F and B (1992) and the related commercial proposals currently available on the market

Figure 1 depicts the two main families of tests and their commercial proposals. The most popular and developed measurement remains that of oxygen decrease either by specific measurement of O_2_ (OECD 301 D, 1992) or by manometry (OECD 301 F, 1992) which directly derives from the method that was developed by Warburg in 1900 (see Perkins, 1943 for a full technical review of all respirometers based on Warburg’s principle). The main advantages of closed systems are the ability to test all types of substances (water soluble, insoluble, and volatile). In the latter test, two modes of monitoring have been developed. The first one is based on the decrease of pressure linked to the consumption of oxygen (by capturing CO_2_) giving rise to a large number of commercial variations (WTW Xylem) and the second one is by reintroducing the consumed oxygen to maintain a constant pressure (Moneratec, Sapromat).

Conversely, the OECD 301 D (1992) test, which specifically measures oxygen, is ultimately not commercially developed. Each laboratory is equipped with BOD-type bottles and specific probes (polarographic or optode). The Skalar company has automated the dilution and preparation procedure, but the incubation of the samples remains separate from the measurement.

**Fig. 2:**
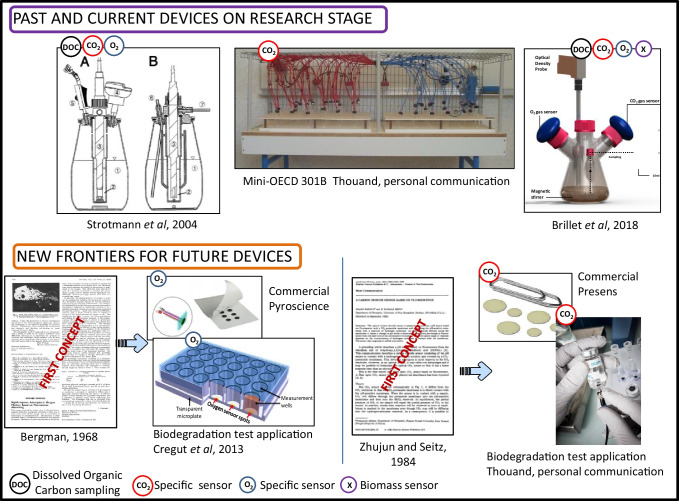
Some devices developed at a research stage and new opto-sensors for O_2_ and CO_2_ measurement illustrating the miniaturization that sensors allow for high-throughput platforms (the first publications depict the period between the discovery of the optodes and the use by the stakeholders)

On the product side of the biodegradation equations, the production of CO_2_ is the parameter of choice to express a mineralization of the substance whether it is insoluble in water or not. It has also to be mentioned that the monitoring of this parameter is equivalent to the estimation of oxygen consumption on the reactant side of the biodegradation equations. Technically, the OECD 301 B (1992) test is based on the developments of Ludzack et al. (1959) which were later improved by Sturm (1973). Since then, nothing has changed, and the system to be put in place remains complex to manage over time, even if it is inexpensive in its design. The commercial device that is closest to the original Sturm method remains the Respicond developed and presented by Nordgren et al (1988). It allows continuous and automatic measurement of CO_2_ by measuring the conductance after its capture in a KOH solution. Thouand et al. (unpublished results) designed a mini-OECD 301 B (1992) with a constant gas flow, but the apparatus with 24 parallel vessels turned out to be really time-consuming (Fig. 2). The other commercial possibilities are based on the volume of CO_2_ trapped or on pressure variations (Fig. 1).

The use of only one indirect biodegradation parameter can give misleading results (Govind et al. 1997, Pitter and Chudoba 1990). This is particularly important for mixtures of substances. There is a better evaluation of ultimate biodegradation when the carbon transformed into biomass is taken into account (Brillet et al., 2018). Commercial systems at this level are very efficient but also very expensive when equipped with devices for monitoring all gases (e.g., Oxymax, see Fig. 1). The system developed by Tronico also makes it possible for the first time to monitor by nephelometry the biomass produced in order to establish a full assessment of biodegradation.

#### Designing future systems

Several devices remain in the research stage but could constitute a good basis for industrial development. Strotmann et al. (1995, 2004) pioneered the design of two families of systems. A combination of different indirect parameters such as the carbon dioxide production and the removal of DOC in a combined DOC/CO_2_ test system (CTS) offers many advantages (Strotmann et al. 1995, Gartiser, 2017, Gartiser, 2022, Norr et al. 2001). The determination of DOC, CO_2_, and BOD in a multicomponent test system (MCTS, Strotmann et al., 2004) or a test based on ultimately transformed organic carbon (UTOC, Brillet et al. 2018) offers even more possibilities (Fig. 2). Test methods based on CO_2_ production by measuring inorganic carbon (IC) via the change of conductivity in a trap with a solution of potassium hydroxide (KOH) or directly by CO_2_ sensors in the headspace enhance the possibilities to automation and high-frequency on-line measurements. In this way, very accurate biodegradation kinetics are obtained. With an additional online determination of the BOD, an MCTS offers even more possibilities. The sampling ports of this test system can furthermore be used to withdraw samples not only for DOC analysis, but also for specific analysis. So the different combined test systems give the chance to determine both, primary and ultimate biodegradability in one system. This versatility makes these methods also suitable for poorly water-soluble substances, polymers, and highly volatile test compounds. An overview of the different combined test methods for RBTs is given in Table 6.

**Table 6:** Modern combined tests on ready biodegradability

Test	Method	Test principle	Remarks
Combined CO_2_/DOC test (CTS)	Combination of OECD 301 A/B (1992), ISO 9439 (1992) and ISO 14593 (1999); Strotmann et al. 1993a; Gartiser et al. 2017; Gartiser et al. 2022)	Static aerobic test simultaneous measurement of DOC removal and CO_2_ production	Non-volatile compounds; versatile test system
Multicomponent test system (MCTS)	Combination of OECD 301 A (1992), OECD 301 B (1992), OECD 301 E (1992), OECD 301 F (1992), OECD 306 (1992), OECD 310 (2014), ISO 7827 (2010), ISO 9439 (1999), ISO 7827 (2010), ISO 9408 (1999), ISO 14593 (1999); (Strotmann et al. 2004)	Static aerobic test, simultaneous measurement of BOD, DOC removal, CO_2_ production, specific analysis and online measurements possible, determination of carbon flow; CO_2_ determination via a conductivity electrode	Volatile and non-volatile compounds; versatile test system
Ultimately transformed organic carbon (UTOC) system	Combination of OECD 301 A (1992), OECD 301 B (1992), OECD 301 E (1992), OECD 301 F (1992), OECD 306 (1992), OECD 310 (2014), ISO 7827 (2010), ISO 9439 (1999), ISO 7827 (2010), ISO 9408 (1999), ISO 14593 (1999﻿); (Brillet et al. 2018)	Static aerobic test system, online measurements, simultaneous measurement of BOD, DOC removal, CO_2_ production, specific analysis also possible, determination of carbon flow; CO_2_ determination via a CO_2_ sensor	Volatile and non-volatile compounds; versatile test system

Another application of such combined test systems using online determination of CO_2_ and BOD lies in the field of complex matrices, like waste water, solid waste, compost, and soil, where analytical procedures are complicated and restricted. Another striking advantage is the fact that in these systems heterotrophic yield coefficients can be estimated which allow an assessment of the flow of the carbon of the test compound either into carbon dioxide during catabolic oxidation processes or into newly formed biomass by anabolic processes (Kappeler and Gujer 1992; Strotmann et al. 1999; Brillet et al. 2018).

With the need to develop new substances and polymers to comply with environmental requirements, both industry and research laboratories are faced with an explosion in the demand for testing. Therefore, new internal screening platforms have to be developed. One of the possible ways is the use of optodes where the presence of gases such as O_2_ or CO_2_ modifies the fluorescence of a ruthenium salt (Bergmann 1968, Zhujun and Seitz, 1984). These mini-sensors are perfectly suited to miniaturization and to the development of high-throughput platforms for intensive use (Fig. 2, Cregut et al., 2014).

However, in several cases, particularly for polymers, mineralization and fragmentation can be followed which may be advantageous to understand the whole process. Infrared or Raman spectroscopy is now generally available. Therefore, Cregut et al. (2013) could follow the biodegradation of commercial polyurethane formulations by combining respirometry and Raman spectroscopy.

#### Probability approach (Probabio)

Besides the technical optimization of the existing RBTs in combined test systems a totally other approach was developed using different inocula from different sites in combination with probability calculations of biodegradability (Thouand et al. 2011, Brillet et al., 2016). This concept aims at evaluating biodegradability and persistence under various environmental conditions and is a practical approach based on a dilution method to estimate the critical inoculum concentration, which still allows biodegradation. The underlying assumption of this concept is to connect the likelihood to inoculate specific microbial degraders from different environmental compartments in a given biodegradation test with the probability of a chemical being biodegraded. It is assumed that “specific degraders” are responsible for the biodegradation of certain test substances and that they can grow better and faster under favorable conditions (Blok 2001). Using a screening platform and a probabilistic approach, the so-called ProbaBio method provides probability profiles of microbial degradation that are obtained under more realistic environmental conditions. It also has to be mentioned that this approach may generate a best-case consideration for maximum biodegradability and that it has to be further evaluated. On the other hand, this concept seems to be straightforward and offers a possibility to improve the concept of RBTs.

#### Automation of RBTs and prediction of biodegradability

As there are many chemicals that have to be tested in RBTs, there is a high demand for the automation of ready biodegradability tests to enable high throughput. Such an attempt was done for aromatic compounds, which can react with 4-nitrobenzenediazonium tetrafluoroboarate (4-NBTFB) (Martin et al. 2017). The spectrophotometric absorbance of a dye used in the test could be related to the concentration of the test compound and used to determine biodegradation. The test has certain limitations as it is only applicable to chemicals which can react with 4-NBTFB, but there are also other potential dyes available for alternative colorimetric test systems (Walker 1987). Another restriction is that this test system is not suitable for volatile compounds or substances with a high bioaccumulation potential, as determined by the partition coefficient between water and octanol (log Pow).

The European chemicals investigation program REACH also recommends the possibility to use QSAR models (quantitative structure-activity relationships, in silico models) to estimate biodegradability. In the past decades, there have been several attempts to predict biodegradability from chemical structures. Loonen et al., (1999) described a partial least squares (PLS) discriminate model for the prediction of the biodegradability of chemicals under standard OECD conditions. They found that long alkyl chain, hydroxy-, ester-, and carboxyl- groups facilitate biodegradation, whereas the presence of one or more aromatic ring systems and halogen substitutes hinder biodegradation. The accuracy of the predictions (“readily biodegradable” vs “not readily biodegradable”) compared with the results from a MITI (I) test (OECD 301 C, 1992) was in the range of 83 to 89%. In another publication different QSAR models such as VEGA, TOPCAT, BIOWIN V.5 and V.6, and START were tested with a dataset of 722 compounds (Pizzo et al. 2013). The three models of VEGA, TOPCAT, and both BIOWIN versions showed the best accuracy for the prediction of “ready biodegradability” vs. “not ready biodegradability” ranging from 81 to 99%. The differences were mainly depending on the data set available. In another study with pre-manufactured notice (PMN) substances, Boethling et al. (2004) found that the BIOWIN models V.3 (linear) and V.6 (non-linear) and model batteries using Bayesian analysis could reduce false positive results in RBTs and inherent tests and, therefore, improve the overall misclassification rate. On the other hand, there is some criticism towards the BIOWIN software, as it does not allow reliable discrimination between readily and non-biodegradable compounds especially for quaternary carbon compounds. Another software (CATALOGIC) is said to have a restricted applicability due to its reduced predictive power (Seyfried and Boschung 2014).

Overall, QSAR programs offer an opportunity to distinguish easily between test compounds, which are likely to be readily biodegradable and those not readily biodegradable. A practical approach might be that compounds with a high likelihood to be readily biodegradable should be tested in simple and inexpensive RBTs, whereas compounds which are unlikely to be readily biodegradable, should directly be tested in more intensive tests like the enhanced (prolonged) RBTs, inherent tests or even in simulation tests. A point to be further approved would be a good applicability catalog of the QSAR programs, as this would offer the chance to a better overall performance.

#### Impact of biodegradation tests on the evaluation of persistence

In the framework of evaluation processes for persistent, bio-accumulating and toxic (PBT) substances ready biodegradability tests of the OECD 301 series and the OECD 310 (2014) (CO_2_ head space test) are used for testing ready biodegradability and to differentiate whether a substance is “not persistent” or “potential persistent” (inconclusive). The conclusion for a substance as being “persistent" cannot be drawn from RBT tests. In contrast, results showing inherent biodegradability ≤ 20% can be used as evidence to conclude that a substance fulfills the criteria for being “persistent” (ECHA 2017b). It has been proposed to apply a similar approach for enhanced RBTs while differentiating between persistent (≤ 20% degradation), “not persistent” (≥ 60% or ≥ 70%, respectively), or “potentially persistent” (> 20% to < 60% or < 70%, respectively) (Gartiser et al. 2022 and 2023; ECHA 2017b). A positive result in one of the RBTs is sufficient for non-persistency, but in case of a negative result, another test system should be used. Among these test systems, all tests are regarded to be equivalent. Difficulties may arise, when contradictory results occur and expert judgment is claimed to be necessary. When there are consistent negative results, an OECD simulation test (OECD 307, 2002; OECD 308, 2002; OECD 309, 2004) should be performed which also allows the assessment of the degradation half-life. These tests are performed under more realistic environmental conditions. The drawback of simulation tests is that they are rather cost and time intensive and normally require the use of ^14^C labeled compounds (Gartiser et al. 2022). It is obvious that there is a gap between the ready biodegradability tests and these simulation tests, which can be closed by the use of eRBTs. The aim is to avoid false negative results that lead to the labeling of persistence. A thorough discussion is given by Kowalczyk et al. 2015, Gartiser et al. (2017, 2022) and in a wider sense taking into account environmentally relevant data by Hughes et al. (2020, 2022a, b).

Furthermore, it has also to be mentioned, that in some special cases, it may be sufficient to determine the elimination of a substance from wastewater without clarifying its degradability or persistence. An example is the removal of dyestuffs in WWTPs by adsorption processes on activated sludge. An appropriate test method was described by Pagga and Taeger (1994).

#### A new challenge: testing of UVCBs and difficult compounds

UVCBs often consist of constituents which are not clearly defined and which may be very complex concerning their composition. Therefore, the assessment of the persistence and biodegradability of such compounds is very demanding. The classical ISO and OECD tests have been designed for pure substances, which can be clearly characterized, but not for mixtures of chemicals and not at all for UVCBs. Biodegradation tests based on carbon dioxide formation, oxygen demand, or DOC decay may be applied to UVCBs, but it is unlikely that the results reflect the persistence of all ingredients, because these mixtures may contain substances between ready biodegradability and persistence (Lai et al. 2022; Hughes et al. 2022a). Simulation tests using ^14^C labeled compounds are not possible for mixtures because every single component would have to be labeled. A way out of this dilemma can be the use of test systems with carbon balances (MCTS: Strotmann et al. 2004, UTOC: Brillet et al. 2018) which might be coupled with a microbial toxicity test (e.g., OECD 209, 2010; ISO 8192, 2007; ISO 9509, 2006; ISO 11348, 2007) at the start and end of the test period in order to calculate a weight of evidence. Also, the annex E of ISO 14851 (2019) respectively annex C of ISO 14852 (2021), provide guidance on how to establish a carbon balance by regarding the total mineralization measured as CO_2_ or BOD, DOC elimination, the increase in biomass, and the determination of the residual test compounds. In such tests, the level of transformed carbon, either mineralized to CO_2_, to catabolites, or integrated into biomass, has to be assessed. This may be further supported by the determination of BOD and DOC removal.

Some UVCBs have simple chemical compositions and appear to be homogenous, for example, gas-to-liquid fuels (GTL) where the measurement of one substance may be representative of the mixture. A recent study (Brown et al. 2020) shows that such a procedure is possible. On the other hand, UVCBs may consist of constituents which are poorly water soluble, toxic, or volatile, resulting in an increase of the technical and analytical effort to determine their biodegradability.

Considering all the known facts, it must be stated that the determination of the persistence of UVCBs is still in the stage of test method development and many analytical and technical challenges have to be solved. An example is the impact of mixture effects, if there are constituents with a stimulating or inhibiting effect on the biodegradation potential of the degrading microorganisms (Lai et al. 2022). Other challenging points are physico-chemical properties such as poor water solubility, hydrophilic and hydrophobic properties, volatility, and adsorbing potential to biomass, which have to be solved before UVCBSs may generally be tested.

Besides UVCBs also other test materials belong to the group of the so-called “difficult compounds” related to biodegradation or persistence assessment. These are compounds which are poorly water soluble, absorb to other chemicals, to the test vessel and biomass, as well as volatile substances. In addition, compounds with a certain toxicity to microorganisms and substances which are ionisable, complexing, unstable, and chiral belong to this group (Hughes et al. 2022b). For some of these compounds (poorly water soluble, volatile ,or toxic), suitable test methods and also a guidance for testing (ISO 10634, 2018—Guidance for poorly water soluble test compounds) already exist. However, for many problematical substances, there is still a need for discussion and test evaluation.

#### Further development of OECD guidelines

Most of the current OECD guidelines have been published in 1992 and are still widely used. In a survey among 16 highly qualified laboratories (mainly GLP certified) from Germany, Switzerland, and the UK the current status and options for improvement have been discussed. The two main RBTs currently applied are the CO_2_ evolution test (OECD 301 B, 1992) and the manometric respirometry test (OECD 301 F, 1992), which account for at least 80% of all RBTs performed, followed by the CO_2_ headspace test (OECD 310, 2014), and the closed bottle test (OECD 301 D, 1992). The DOC-based tests (OECD 301 A and E, 1992), the MITI (I) test (OECD 301 C, 1992), and the marine tests (OECD 306, 1992) have a minor importance.

About half of the laboratories suggested omitting the DOC-based tests because of the unspecific endpoint DOC. Others suggested to merge the OECD 301 A and E (1992) tests into one method. About half of the laboratories also indicated that the MITI (I) test (OECD 301 C, 1992) could be withdrawn due to the effort for cultivating the inoculum and its low inoculum potential. Others suggested to merge the OECD 301 C and F (1992), both being respirometric methods. The acceptance of activated sludge from municipal WWTPs instead of the artificial MITI sludge would certainly promote the performance of the MITI (I) and MITI (II) tests (OECD 301 C (1992) and OECD 302 C (1981)). Most laboratories supported the use of any of the allowed OECD 301 inoculum sources in any RBT as far as the validity criteria are met. However, the signal-to-noise ratio should be considered. Six out of 16 laboratories were also open to merge different testing principles by establishing combination tests with several endpoints, such as CO_2_ evolution combined with oxygen consumption, CO_2_ combined with DOC, next to oxygen consumption combined with DOC, or all three different endpoints. However, a minor part of laboratories was against the obligatory multi-end-point testing due to the equipment needed and the different substrate/inoculum ratios required for accurate testing. In addition, a minor part referred to difficulties in handling results obtained from different monitored parameters. Details of this survey are documented by Gartiser et al. (2023).

#### Applications in environmental biotechnology

Standardized tests are not only used to test chemicals for biodegradation purposes, but can also be used also in modified forms in other fields of environmental biotechnology as pre-tests to evaluate possible biological treatment processes or even to design the layout of real treatment plants. Applications can be found in WWTPs, in biological air treatment (bio-filters and bio-washers), and in the field of treatment of contaminated soils. An example of the latter important application field is here shortly outlined.

Since the very beginning of the modern chemical economy, there were always attempts of microbiologists to detect, isolate, and characterize microorganisms that have the capability to degrade these chemicals. This also led to the implementation of a whole industry dealing with the bioremediation of sites contaminated with recalcitrant chemicals. Pre-tests based on specifically modified standardized test methods offer important tools to evaluate the possibility of a biological treatment method. In case of a positive result, two main strategies can be applied. The most basic engineered bioremediation techniques, namely biostimulation, relies on the application of nutrients, terminal electron acceptors, and additives, which stimulate the activity of native degrading microorganisms, as they are present in every site. On the other hand, bioaugmentation is based on the introduction of selected microbial species specialized in the biodegradation of specific compounds directly into the contaminated site. While the idea is potentially promising, investigations focused on the practical application of bioaugmentation revealed several flaws. Bioaugmentation seems only to be applicable in cases of very specific pollution and/or specific environmental conditions and when pollutants are present in high concentrations (Atashgahi et al. 2018, Lawniczak et al. 2020).

The use of isotopically labeled (e.g., ^13^C or ^15^N) chemicals in biodegradation tests enables the detection of microbial activity by tracking the label in different biomolecules (fatty acids, carbohydrates, DNA, RNA, and proteins) of the organisms (Martinez-Lavanchy et al. 2015, Wang et al. 2016). Here, the most recent implementation of protein-based stable isotope probing (protein-SIP) allows to link functional and phylogenetic information provided by peptide identification with the detection of substrate utilization as a proxy for metabolic activity (Luensmann et al. 2016, Jehmlich et al., 2016).

#### Proof of pollutant (plastic) degradation in laboratory and environment

To date, global plastic production is close to 400 million tons per year, and the overall amount of plastic waste on earth is about 7.300.000.000 tons mainly caused by polyethylene (PE, 36%); polypropylene (PP, 21%); polyvinyl chloride (PVC, 12%); polyethylene terephthalate (PET, 10%), polyurethane (PU, 10%), and polystyrene (PS, 10%) (PlasticsEurope 2019). Most plastic materials are very durable in the environment, but there are some which are biodegradable. Thus, it is important to identify the latter and to assess the rate and the degree of biodegradation. For this purpose, standardized tests offer usable and reliable tools (ISO 14851, 2019, ISO 14852, 2021; ISO 14851-1, 2012; ISO 14851-2, 2018). More detailed information can be found in the literature (e.g., Cardenas Espinosa et al. 2020, 2021). For a sustainable future of plastics, it is important to produce more biodegradable plastics and to organize better recycling of all plastic materials. It is conceivable that industry will use more degradable precursors in the future production of plastics, taking into consideration scientific knowledge about the biodegradability of these precursors (Puiggene et al. 2022). However, a fundamental switch to new plastic compounds is only achievable when changes in the governance and legislation of plastic materials take place. In the European Union, this mainly concerns widespread plastics such as packaging materials.

The biodegradability of plastics may depend on the chemical compounds that make up the plastic. Generally, polyesters such as polyethylene terephthalate (PET) and polyester polyurethane (PU) can be hydrolyzed by enzymes that also degrade naturally occurring polymers in plants and animals. In contrast, plastics such as polyethylene (PE), polypropylene (PP), polystyrene (PS), and polyvinyl chloride (PVC), which mainly consist of stable C-C bonds, and polyether polyurethane (PU) are very difficult to degrade. They are broken down into small particles mechanically or by UV light, but are practically biologically inaccessible. The most important research question for environmental microbiologists working on this topic is which plastics can be attacked by microorganisms at all. This information can be helpful both for the industry with regard to the chemical structure of future plastic products and for legislation (e.g., local governments or the EU Commission) with regard to the future banning of certain types of plastics. This mainly concerns plastics for single use with a big possibility to enter the environment after use. At present, there already exist plastic bin liners that decompose under controlled composting conditions and can, therefore, be easily used in households. But up to now, the number of possible applications is insufficient, and for this reason, clear definitions of degradability including limit values and more reliable test methods need to be implemented.

### Final remarks

In the course of intensive discussions about climate change and environmental pollution, chemicals are increasingly coming into the focus of experts and the public. With regard to maintaining an intact environment, a lot has already been achieved in recent years, but there is still plenty to do. Consequently, recalcitrant chemicals should be avoided from the biosphere. The biodegradability of substances, especially anthropogenic chemicals, is a fundamental factor in these considerations, since a substance that disappears from the environment is potentially less dangerous than a persistent one.

As this review shows, there are already a number of useful test methods and investigation strategies with regard to biodegradability, but it also shows that there are still many shortcomings and that more work has to be done by society, especially by the authorities, science, and the industry. Not only do test methods help to detect problems in the environment or predict the behavior of chemicals, but they are also of great use in the development of new, environmentally friendly products. Against this background and supported by the effort to maintain a healthy environment, the constant further development of the tools used, in this case, efficient biodegradation tests, is of great importance for all of us, and these tests will be an integrative part of sustainable chemistry with a circular chemical economy.

## Supplementary information

Supplementary material

## Data Availability

All data generated or analyzed during this study are included in this published article (and its supplementary information files).
