# Supplementary material for: Toward the future of OECD/ISO biodegradability testing-new approaches and developments
Source: Appl Microbiol Biotechnol. 2023 Mar 3;107(7-8):2073–95. doi: 10.1007/s00253-023-12406-6 (PMC10033483; doi:10.1007/s00253-023-12406-6)
Supplement: Supplementary file 1 — Supplementary material [file 253_2023_12406_MOESM1_ESM.pdf]

Supplementary Material

Toward the future of OECD/ISO biodegradability testing - new approaches and developments

Uwe Strotmann[1], Gerald Thouand[2], Udo Pagga[3], Stefan Gartiser[4] and Hermann J. Heipieper[5]*

[1]Westfälische Hochschule, Dept. of Chemistry, Recklinghausen, Germany (uwe.strotmann@w-hs.de)

[2]Nantes Université, ONIRIS, CNRS, GEPEA, UMR 6144, 85000 La Roche sur Yon, France, France, (gerald.thouand@univ-nantes.fr)

[3] Rüdigerstr. 49, 67069 Ludwigshafen, Germany (udo.pagga@t-online.de)

[4] Hydrotox GmbH, Bötzinger Str. 29, 79111 Freiburg, Germany (gartiser@hydrotox.de)

[5]Department of Environmental Biotechnology, Helmholtz Centre for Environmental Research - UFZ, Leipzig, Germany (hermann.heipieper@ufz.de)

*corresponding author: Hermann J. Heipieper, hermann.heipieper@ufz.de

Table S1. Data about existing chemicals (modified after Wang et al., 2020)

Number of chemicals and mixtures of chemicals registered for production and use:<br>- claimed confidential<br>- ambiguously described	> 350,000<br>> 50,000<br>up to 70,000
Total chemicals with CAS numbers<br>- individual compounds<br>- organics<br>- mixtures, polymers, UVCBs	235,323<br>157,896<br>137,325<br>75,384

Table S2. Microbial toxicity tests

Test	Method	Test principle
Inhibition of heterotrophic respiration	OECD 209<br>ISO 8192	measurement of oxygen consumption due to heterotrophic respiration
Inhibition of nitrification inhibition	OECD 209<br>ISO 9509	measurement of oxygen consumption due to nitrification respiration (OECD 209) or ammonium oxidation, nitrite and nitrate formation (ISO 9509)
Luminescent bacteria test	ISO 11348 part 1 to 3	measurement of inhibition of light emission
Pseudomonas putida growth inhibition test	ISO 10712	measurement of growth inhibition of a growing pure culture of *Pseudomonas putida*
Growth inhibition test with activated sludge bacteria	ISO 15522	measurement of growth inhibition of activated sludge bacteria
Determination of the inhibition of the activity of anaerobic bacteria	OECD 224<br>ISO 13641	measurement of inhibition of biogas production

Table S3. Bin system of reference compounds (modified after Comber and Holt, 2007)

There are four bins for classification:
Bin 1: normally passes a RBT or modified RBT
Bin 2: normally passes an enhanced RBT, but currently fails any other RBT
Bin 3: normally fails any RBT
Bin 4: should never pass an enhanced or modified RBT

Classification	Compounds
Bin 1	Aniline, sodium benzoate, 1-octanol, anthraquinone, phenol
Bin 2	Diethylene glycole, 4-chloroaniline, 1,3,5-trimethylbenzene, Dinitrotoluene, 4-Fluorophenol
Bin 3	Di-isotridecyladipate, Cyclododecane, Cyclododeca-1,5,9- triene , Cyclododecane , Dibutylphenol
Bin 4	Musk xylene , Hexachlorobenzene, Benzo(a)pyrene, Hexachlorohexane

Table S4. Biodegradation of some commonly used reference compounds recommended by the OECD

Compound	Test system	Extent of degradation (%)	References
Benzoic acid, sodium benzoate	OECD 301 F	91 - 93	Reuschenbach et al., 2003; Gu et al., 2020
Aniline	OECD 301 A - F<br><br>MCTS*<br>- DOC elimination<br>- oxygen consumption<br>- CO_2 production<br><br>CTS**<br>- DOC elimination<br>- CO_2 production<br><br>OECD 302 B	75 - 98<br><br><br>96<br>82.5<br>83<br><br><br>> 95<br>78<br><br>98	Reuschenbach et al., 2003, Strotmann, et al., (2004), Gu et al., 2020<br><br>Strotmann et al., 2004<br><br>Strotmann et al., 1995<br><br>Reuschenbach et al., 2003
Diethylene glycole	OECD 301 F<br><br>CTS**<br>- DOC elimination<br>- CO_2 production<br><br>OECD 302 B	59 - 93<br><br><br>> 95 - 98<br>90 - 99<br><br>70	Reuschenbach et al, 2003; Gartiser et al., 2022<br><br>Strotmann et al., 1995, Gartiser et al., 2022<br><br>Comber et al., (2010); Lapertot et al., 2006
Sodium acetate	OECD 301 F	> 95	Gu et al., 2020
Sodium benzoate	OECD 301 F	> 95	Gu et al., 2020
1-octanol***	OECD 310	85	Comber et al., 2010; Battersby et al., 1997

* multi component test system based on OECD 301 tests; ** combined test system based on OECD 301 A/B tests; *** water insoluble compound
